# Diffuse Large B-Cell Lymphoma With a Background of Extensive Granulomatous Inflammation: A Potential Pitfall for Misdiagnosis

**DOI:** 10.7759/cureus.16198

**Published:** 2021-07-05

**Authors:** Shakiba Hassanzadeh, Nicholas Mackrides, Shima Rastegar, Reza Nejati

**Affiliations:** 1 Medicine, Isfahan University of Medical Sciences, Isfahan, IRN; 2 Hematopathology, Fox Chase Cancer Center, Philadelphia, USA; 3 Pathology, Rutgers New Jersey Medical School, Newark, USA; 4 Pathology/Hematopathology, Fox Chase Cancer Center, Philadelphia, USA

**Keywords:** granuloma, lymphoma, potential pitfall for misdiagnosis, diffuse large b-cell lymphoma, granulomatous inflammation, diffuse large b-cell lymphoma with granulomatous inflammation

## Abstract

Granulomatous inflammation has been reported to be associated with Hodgkin and non-Hodgkin lymphomas. Here, we report a case of recurrent diffuse large B-cell lymphoma (DLBCL) with extensive granulomatous inflammation that was initially misdiagnosed as granulomatous lymphadenitis. In 2019, a 75-year-old Caucasian male presented to our hospital with an enlarged right supraclavicular lymph node. He had a medical history of prostate cancer (in 2004), DLBCL (initially diagnosed in 2009), and rectal adenocarcinoma (in 2017), all of which responded well to treatment. In 2018, the patient had experienced right axillary adenopathy, weight loss, and intermittent night sweats. An excisional biopsy of a right axillary lymph node, performed at another institution, was diagnosed as granulomatous lymphadenitis. In 2019, at our hospital, an excisional biopsy of a right supraclavicular lymph node showed DLBCL in a background of granulomatous inflammation. A review of the prior right axillary lymph node biopsy also showed DLBCL with a background of extensive granulomatous inflammation. Chemotherapy was initiated and the patient’s follow-up showed a good response. We report this case to raise awareness that granulomatous inflammation may obscure the diagnosis of some neoplasms, such as DLBCL, which are less commonly known to have granulomatous inflammation. This may result in delayed treatment and may ultimately affect outcomes.

## Introduction

Diffuse large B-cell lymphoma-not otherwise specified (DLBCL-NOS) (referred to as DLBCL in this study) is the most common subtype of non-Hodgkin lymphoma (NHL) and has an incidence rate of 6.3 per 100,000 in the USA. It is more common in males compared to females and in the elderly population [1,2].

The exact etiology of DLBCL is still unknown. Although these neoplasms are usually de novo, they may also occur secondary to the transformation of a less aggressive B-cell lymphoma. Immunodeficiency is a risk factor for DLBCL, and such cases are more often Epstein-Barr virus-positive than sporadic cases. Patients with DLBCL often present with the rapid growth of tumors at one or more nodes or extranodal organs. Any extranodal organ may be involved as the primary site of DLBCL, but the gastrointestinal tract is the most common site. Furthermore, patients with DLBCL may be asymptomatic, have B symptoms, or have localizing symptoms (depending on the site of the extranodal involvement) [2,3].

According to the fourth revision of the World Health Organization classification for lymphomas in 2016, DLBCL is a neoplasm of medium or large B lymphoid cells with diffuse growth patterns. The nuclei of these cells are of the same size or larger compared to those of normal macrophages or are more than twice the size of those of normal lymphocytes. Furthermore, these cells may express B-cell markers such as CD19, CD20, CD22, CD79a, and PAX5 [2,4]. DLBCL is heterogeneous in its clinical, morphological, pathological, and molecular features. It has different morphologic variants, including centroblastic, immunoblastic, anaplastic, and other rare variants [2,5].

Chronic granulomatous inflammation may be caused by various conditions, such as infections, autoimmune disorders, toxins, allergens, and neoplasms. It is characterized by aggregates of histiocytes (macrophages), which appear epithelioid, with round-to-oval nuclei and granular eosinophilic cytoplasm. Additionally, the merging of these cells forms multinucleated giant cells [6]. Epithelioid granulomas have been reported to be associated with various malignant solid tumors and lymphomas, including Hodgkin lymphoma (HL) (more common) and various types of NHL [7]. However, there is limited knowledge and scarce data about chronic granulomatous inflammation associated with DLBCL [7,8]. In this study, we report a case of recurrent DLBCL with extensive granulomatous inflammation which was initially misdiagnosed and treated as granulomatous lymphadenitis.

## Case presentation

A 75-year-old Caucasian male presented to our hospital with an enlarged right supraclavicular lymph node in January 2019. He had a history of localized prostate cancer (Gleason score 6) in 2004 that was treated with brachytherapy. In 2009, the patient presented with adenopathy, and a biopsy of a right axillary lymph node showed DLBCL. Flow cytometry detected B-cells with a predominance of lambda light chain expression. A positron emission tomography (PET) scan showed extensive fluorodeoxyglucose (FDG)-avid lymphadenopathy above and below the diaphragm, stage IV, with biopsy-proven bone marrow involvement. He was treated with six cycles of rituximab, cyclophosphamide, doxorubicin, vincristine, and prednisone with complete response. In addition, he was diagnosed with rectal adenocarcinoma in 2017 and was treated with neoadjuvant chemoradiation followed by pelvic exenteration. The patient had no history of tuberculosis, sarcoidosis, or other diseases associated with granulomatous inflammation.

**Figure 1:**
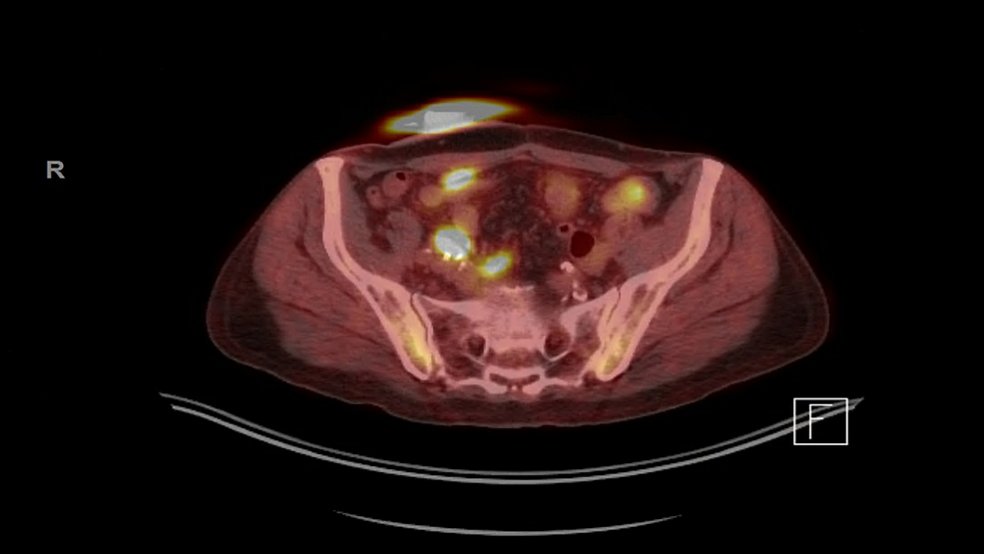
PET/CT image showing multifocal FDG-avid lesions. PET/CT: positron emission tomography/computed tomography; FDG: fluorodeoxyglucose

In 2018, the patient experienced right axillary adenopathy, weight loss, and intermittent night sweats. An axillary lymph node biopsy performed at an outside institution was diagnosed as granulomatous lymphadenitis. Flow cytometry was not performed on tissue from the biopsy. His PET scan showed multifocal FDG-avid soft tissue lesions, lymphadenopathy, and osseous lesions (Figure 1).

**Figure 2:**
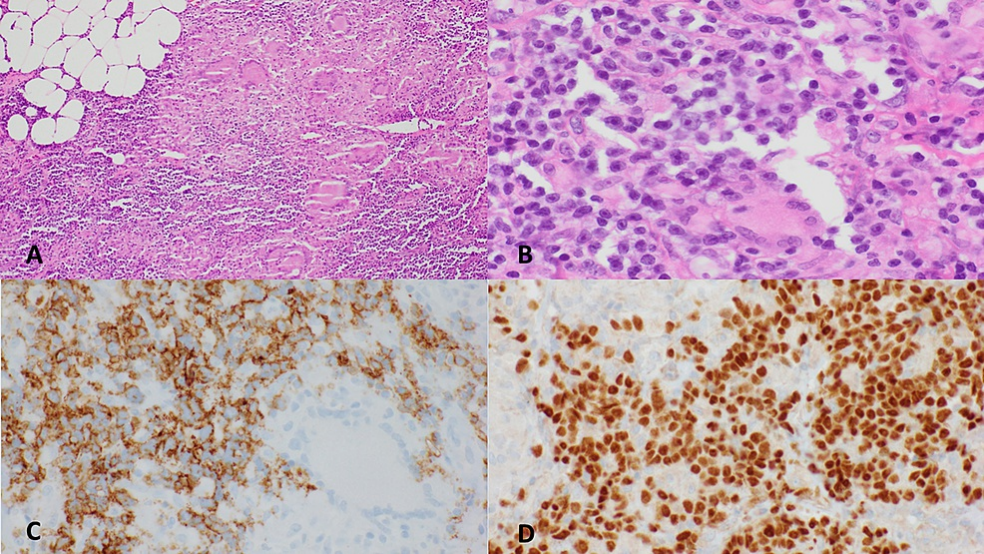
Lymph node biopsy. Lymph node biopsy showing extensive non-necrotizing granulomatous inflammation associated with patchy aggregates of medium-to-large atypical lymphocytes (A: 40×; B: 100×). (C) Immunostaining for CD20 highlights the clusters of large cells. (D) Lymphoma cells show nuclear staining for PAX5.

In January 2019, a biopsy of the patient’s enlarged right supraclavicular lymph node was performed, which showed DLBCL in a background of granulomatous inflammation (Figure 2). Flow cytometry detected a population of lambda restricted B-cells. The prior right axillary lymph node biopsy (2018) was then reviewed at our institution and showed DLBCL with a background of extensive granulomatous inflammation.

Therefore, therapy with rituximab, cyclophosphamide, etoposide, vincristine, and prednisone was started in February 2019. A follow-up PET scan in April 2019 showed minimal partial response, and a biopsy of a PET-avid right axillary lesion confirmed residual DLBCL with extensive granulomatous inflammation. Therefore, the therapy was switched to rituximab, gemcitabine, cisplatin, and dexamethasone in May 2019, which showed partial response with very little residual disease on PET/CT. Treatment was not well tolerated. Consequently, polatuzumab, Rituxan, and bendamustine were initiated in January 2020, but were also not well tolerated; therefore, they were discontinued in May 2020. A follow-up PET/CT in July 2020 showed a very good response.

## Discussion

Different types of NHLs have been reported to be associated with concurrent granulomatous inflammation, including follicular lymphoma, small lymphocytic lymphoma, and different types of T-cell NHLs [9]. Currently, there is limited data on the association of DLBCL with granulomatous inflammation [8]. Although the exact cause of granulomatous inflammation in NHLs is still unknown, there have been several suggestions for the mechanisms of granuloma formation. For instance, T-helper cells cause a hypersensitivity reaction to the antigens produced by tumor cells, and this, in turn, activates the monocytes which results in the production of epithelioid histiocytes [10,11]. In addition, aberrant cytokine production may be another mechanism for the production of epithelioid histiocytes [8,12].

Nonetheless, it has been discussed that, occasionally, epithelioid granulomas may obscure the underlying conditions on fine-needle aspiration cytology assessment and cause problems in diagnosing and differentiating granulomas and neoplasms that cause granulomas. Khurana et al. presented six cases that had granulomatous inflammation on cytology; however, on histological findings, they were diagnosed with HL, lymphoepithelial carcinoma, DLBCL, anaplastic carcinoma, and squamous cell carcinoma of the larynx. Therefore, they emphasized the importance of excisional biopsy in cases when it is difficult to diagnose solely based on cytology results, especially when the clinical features suggest malignancy [7].

In 2016, Nyunt et al. reported a case of DLBCL associated with chronic granulomatous inflammation. This patient was initially misdiagnosed as tuberculosis (TB) as her first biopsy showed chronic granulomatous inflammation suggestive of TB. She was treated with antitubercular treatment for two months without any improvement in her symptoms; therefore, another biopsy was performed which showed DLBCL. Consequently, chemotherapy with rituximab, etoposide, vincristine, doxorubicin, cyclophosphamide, and prednisone was initiated and she responded well to this treatment [8]. Moreover, in 2018, Yucesoy et al. reported a case of mandibular DLBCL with metastasis of the gastrointestinal tract. The patient initially presented with a mandibular lesion and the first biopsy showed peripheral giant cell granuloma. However, upon further assessment, a second biopsy revealed DLBCL [13].

Similarly, in our case, in 2018, the initial outside review of the right axillary lymph node showed granulomatous lymphadenitis. However, on further assessment in 2019, a biopsy of his right supraclavicular lymph node showed DLBCL associated with granulomatous inflammation. Consequently, the right axillary lymph node biopsy was re-reviewed which revealed DLBCL with extensive granulomatous inflammation. Therefore, chemotherapy for recurrent DLBCL was initiated.

Various factors affect the prognosis in DLBCL such as age, stage, performance status, extranodal involvement, and lactate dehydrogenase serum level, among others [2]. In few reports, it has been proposed that granulomas in HL or NHL may be associated with a more favorable prognosis [10,14]. However, whether granulomatous inflammation has any effect on the prognosis of DLBCL is still unclear and requires further investigation.

## Conclusions

Diagnosing DLBCL can be challenging, especially when there is extensive granulomatous inflammation, which may obscure malignant cells and potentially lead to misdiagnosis. This, in turn, may affect the patient’s prognosis, proper treatment, morbidity, and mortality. Therefore, it is important to consider neoplasms, such as DLBCL, as a differential diagnosis when encountering granulomatous inflammation on histopathology.
